# A Multi-Phasic Perspective of the Intact Permeability of the Heterogeneous Argillaceous Cobourg Limestone

**DOI:** 10.1038/s41598-019-53343-7

**Published:** 2019-11-22

**Authors:** A. P. S. Selvadurai

**Affiliations:** Department of Civil Engineering and Applied Mechanics, McGill University, Montréal, QC H3A 0C3 Canada

**Keywords:** Environmental sciences, Hydrology

## Abstract

The Cobourg limestone is a heterogeneous argillaceous rock consisting of *lighter* nodular regions of calcite and dolomite, interspersed with *darker* regions composed of calcite, dolomite, quartz and a clay fraction. The intact permeability of the Cobourg limestone is estimated to be in the range of *K* ∈ (10^−23^, 10^−19^) m^2^. This paper discusses the factors influencing the measurement of the intact permeability of the Cobourg limestone and presents an upscaling approach for estimating this parameter. The procedure first involves the dissection of a cuboidal sample of the rock measuring, 80 mm × 120 mm × 300 mm, into ten 8 mm-thick slabs. Digital imaging and mapping of the larger surfaces of these sections are used to create, from both surface image extrusion and surface image interpolation techniques, the fabric within the dissected regions. The estimated permeabilities of the lighter and darker regions are used in the computational models of the computer-generated fabric to estimate the effective permeability of the rock. These results are complemented by estimates derived from mathematical theories for estimating permeabilities of multiphasic composites.

## Introduction

**Figure 1:**
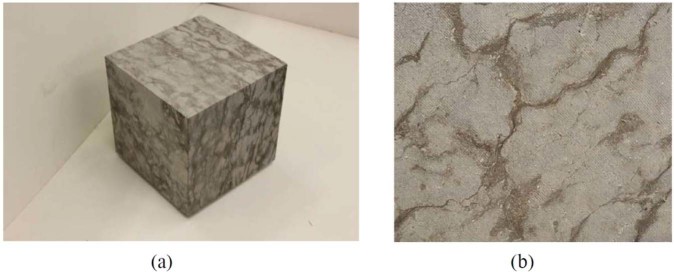
The fabric of the Cobourg limestone. (**a**) The *lighter* nodular features in a 300 mm cuboid (**b**) the *darker* argillaceous partings over a 100 mm × 120 mm area.

The Cobourg limestone formation occurs across southeastern Ontario within the Paleozoic sedimentary sequence that rests on a Pre-Cambrian basement of the granitic rock forming the Canadian Shield. Interest in this limestone stems from its potential suitability as a host rock for a Deep Geologic Repository (DGR) to be sited near the eastern shores of Lake Huron in southern Ontario, Canada^1^, for storing low- and intermediate-level, non-heat emitting radioactive waste. The proposals for the DGR call for its siting to be approximately 680 m below ground level within the Cobourg limestone formation. The Cobourg limestone host rock is overlain by Upper Ordovician-age siltstone and gray shale extending to a thickness of approximately 200 m and underlain by argillaceous limestone and gray shale, approximately 150 m thick. Despite its low clay content, the Cobourg limestone is nominally referred to as an *argillaceous limestone*. A characteristic visual feature of the Cobourg limestone is the extraordinary heterogeneity of the fabric, consisting of *lighter* nodular regions of calcite and dolomite separated by *darker* argillaceous partings of a similar composition but with quartz and a low clay content (Fig. 1).

The mineralogical compositions obtained from XRD techniques performed at the McGill Institute for Advanced Materials are summarized for completeness: (i) the lighter grey nodular phase consists of: *Calcite* ~82.4%; *Dolomite* ~1.3%; *Quartz* ~7.5%; *Clay* ~ 8.8%, with a Porosity ~ 0.001; (ii) the darker argillaceous partings consist of: *Calcite* ~ 69.5%; *Dolomite* ~ 4.2%; *Quartz* ~ 16.9%; *Clay* 9.4%, with a Porosity ~ 0.006. ~ In the aforementioned, the clay fraction includes varying proportions of Illite, Kaolinite and Montmorillonite. The partings give the appearance of a distinct heterogeneity and on occasions, the presence of nominal stratifications and raises important issues related to the dimensions of the representative volume elements (RVE) that should be tested to obtain meaningful intact permeability estimates, necessary for assessment of a DGR constructed in the Cobourg formation. SEM images of the Cobourg limestone distinctly indicate the presence of the calcite phase, the argillaceous phase, the micro-texture boundary and a well-developed micro-porosity network in the argillaceous phase that contributes to the higher porosity. Previous studies^2,3^ indicate that a suitable REV for the Cobourg limestone should be a cuboidal region on the order of 75 mm in dimension.

**Figure 2:**
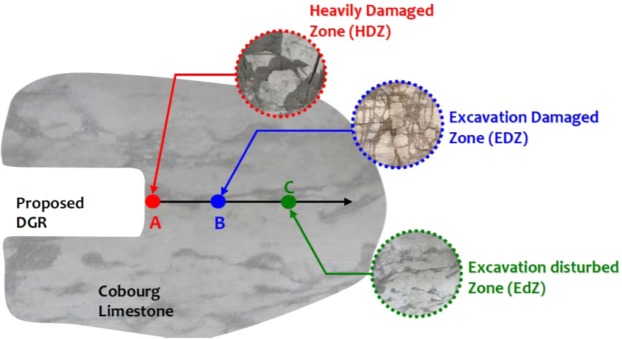
Schematic representation of the potential development of damage around an underground opening in the Cobourg limestone^13^.

It is understood that the creation of a DGR will perturb the geostatic stress state at the repository location and will lead to the creation of Highly Damaged Zones (HDZ), Excavation Damaged Zones (EDZ) and Excavation disturbed Zones (EdZ), all with altered permeability characteristics due to the generation of visible cracks, micro-cracks and other defects at various scales (Fig. 2). The HDZ, EDZ and EdZ zones shown in Fig. 2 are schematic and not to any scale. These are meant to illustrate qualitatively the influence of the stress states in the vicinity of the DGR on the alteration of the condition of the rock mass. The exact determination of the zones will require a precise specification of the geometry of the DGR opening, the *in situ* stress state and the geomechanical properties of the rock in terms of its deformability, damage in terms of micro-crack development, failure, fracture and movement of discontinuous regions. In the HDZ zone, the rock can experience gross fracturing leading to a fragmented medium. In the EDZ zone there can be micro-crack development that can lead to alterations of the geomechanical and transport properties. In the EdZ zone, the rock is largely undisturbed and the permeability of this region is of interest to modelling the long-term containment that can be provided by the undisturbed rock mass.

When the long-term extent of these damaged zones are established and designated as zones of compromised retardation potential for the migration of radionuclides, the primary natural geological barrier that will provide for the retardation of any released radionuclides from the stored waste to the environment is the intact Cobourg limestone. In this sense, the fluid transport characteristics of *intact* Cobourg limestone is a critical parameter in a concept assessment exercise.

Earlier investigations of the permeability of the Cobourg limestone (also referred to as the Lindsay limestone) are due to Raven *et al*.^4^, Golder Associates^5^, Intera^6^, NWMO^1^, Selvadurai^2^ Mazurek^7^ who used both laboratory steady state tests and *in situ* hydraulic pulse tests to estimate the permeability. Vilks and Miller^8^ conducted steady state axial flow permeability tests on samples measuring 25 mm in diameter and 10 mm in length. This sample size is appropriate if the presence of fabric heterogeneities can be disregarded. The experiments reported by Selvadurai *et al*.^9^ were performed on Cobourg samples measuring 108 mm in diameter and 200 mm in length. The influence of isotropic stress increase and isotropic stress cycling up to 20 MPa, on permeability evolution was investigated. The permeability was estimated using hydraulic pulse tests. The study showed that, in contrast to the conventional reduction in permeability with increasing isotropic compression, the heterogeneous fabric contributed to an increase in the residual permeability (i.e., irreversible permeability change resulting from a loading-unloading cycles. For the two samples tested^9^, the initial permeability at a confining pressure of 5 MPa ranged between (0.2, 1.7) × 10^−22^ m^2^. When the confining pressure was increased to 20 MPa, the permeability in the two samples increased respectively in the range (1.9, 5.9) × 10^−22^ m^2^. When the confining pressure was reduced to 5 MPa, the permeability increased to the range (3.5, 8.7) × 10^−22^ m^2^). Selvadurai and Jenner^10^ conducted both steady state and transient radial flow hydraulic pulse tests on unstressed Cobourg cylinders measuring 106.8 mm in diameter with lengths between 116.8 mm and 173.6 mm. The permeabilities were estimated to be 0.01 × 10^−19^ m^2^ to 2.0 × 10^−19^ m^2^. Nasseri *et al*.^11^ performed Thermo-Hydro-Mechanical (THM) experiments on Coburg limestone samples measuring 50 mm in diameter and 125 mm in length. Hydraulic pulse tests were performed on the samples to determine the permeability of the Cobourg at room temperature, with the estimates varying from 0.117 × 10^−19^ m^2^ to 1.68 × 10^−19^ m^2^. Selvadurai and Najari^12^ present results of permeability tests conducted on unstressed Cobourg cylinders measuring 150 mm in diameter and 278 mm in length containing a 26 mm diameter cylindrical cavity drilled to a depth of 39 mm from a plane surface. When the pressurized cavity is aligned normal to the nominal partings, the estimated permeability ranged from 0.15 × 10^−19^ m^2^ to 1.3 × 10^−19^ m^2^. When the pressurized cavity was aligned along the nominal partings, the estimated permeability ranged from 1.04 × 10^−19^ m^2^ to 3.9 × 10^−19^ m^2^. These results are presented in Table 1. Permeability tests were carried out using both steady state and hydraulic pulse test applicable to the axisymmetric three-dimensional configuration. Selvadurai and Głowacki^13^ performed hydraulic pulse tests on 85 mm diameter and either 86 mm or 127 mm long samples of the Cobourg limestone, subjected to stress states that can lead to the development of damage. The experiments were performed using an Obert-Hoek Triaxial Cell and an MTS Rock Testing Machine to control the deformations. Similar to the experiments reported by Selvadurai *et al*.^9^, the study by Selvadurai and Głowacki^13^ also points to increases in permeability evolution during the application of confining stresses up to 30 MPa and a permanent increase in the permeability upon unloading of the sample to 5 MPa.

**Table 1:** Estimates for permeability of the Cobourg limestone.

Reference	Sample Site Location	*K*_min_ (m^2^) 10^19^	*K*_max_ (m^2^) 10^19^	*K*_median_ (m^2^) 10^19^
Raven *et al*.^4^	OHD-1 Mississauga/Lakeview	0.102	6.42	3.26
Raven *et al*.^4^	UN-2Darlington/ Bowmanville	0.064	13.6	8.19
Golder Assoc^5^	DDH 01/02	1.33	40.8	21.1
Mazurek^7^	*In Situ* Packer Tests in Vertical and Inclined Boreholes	0.0642	40.0	N/A
Vilks and Miller^8^	Tests Perpendicular to the Bedding Plane	<0.001	0.0035	N/A
Vilks and Miller^8^	Tests Along the Bedding Plane	0.0012	0.022	N/A
NWMO^1^	Tests Perpendicular to the Bedding Plane	N/A	N/A	0.979
NWMO^1^	Tests Along the Bedding Plane	N/A	N/A	9.80
Selvadurai *et al*.^9^	Tests Perpendicular to the Bedding Plane	0.0002	0.009	N/A
Selvadurai and Jenner^10^	Tests Along the Bedding Plane	0.01	2.00	N/A
Nasseri *et al*.^11^	Sample orientation unspecified	0.117	1.68	N/A
Selvadurai and Najari^12^	Cavity flow tests with cavity Perpendicular to Bedding plane	0.15	1.3	N/A
Selvadurai and Najari^12^	Cavity flow tests with cavity Along to Bedding plane	1.04	3.9	N/A

Selvadurai and Najari^14^ conducted boundary heating of the experimental configuration to determine the pore pressure generation and decay within the fluid-filled cavity. The cavity pressure rise due to the advancing heat pulse and the subsequent decay can be used to investigate the influence of the Cobourg permeability on the cavity pressure decay response in particular. In a recent investigation by Selvadurai and Głowacki^15^ the local permeability measurements were obtained using miniature flow entry points to determine the permeability of the lighter and darker facies of the Cobourg limestone. Transient pulse tests and steady state tests were performed to estimate the permeability. The results in Table 3 of^15^, contain a range of estimates for the permeabilities of the lighter and darker facies. The steady state tests for these vary from 0.27 × 10^−19^ m^2^ to 8.0 × 10^−19^ m^2^ for the darker material and 0.62 × 10^−19^ m^2^ to 6.3 × 10^−19^ m^2^ for the lighter material.

For low permeability rocks (i.e. permeability in the range 10^−23^ m^2^ to 10^−19^ m^2^), the preferred method for estimating permeability is the hydraulic pulse test. The appeal of this method is the rapidity with which the test can be performed to obtain permeability estimates. The hydraulic pulse method is, however, not without its limitations. When steady state flow is used to estimate the permeability of a saturated rock, the only known information relates to the hydraulic potential difference on two surfaces of the sample, the steady flow rate established by the potential difference, the temperature at which the experiment is conducted and the dimensions of the flow domain. In contrast, for hydraulic pulse tests several additional parameters, including the compressibility of the solid material composing the porous fabric, the compressibility of the porous skeleton, the porosity of the rock and the compressibility of the pore fluid, are required to accurately interpret the results. In addition, factors such as saturation of the sample, the presence of air voids both within the pore space of the sample and within the connections, and the residual pressures present in the sample can influence the results of hydraulic pulse tests conducted on low permeability rocks similar to the Cobourg limestone. These aspects are discussed by Selvadurai and Głowacki^13,15^ and Selvadurai and Najari^16^. The bulk permeability estimates for the Cobourg limestone available in the literature are summarized in Table 1.

## Mapping of the Fabric of the Cobourg Limestone

**Figure 3:**
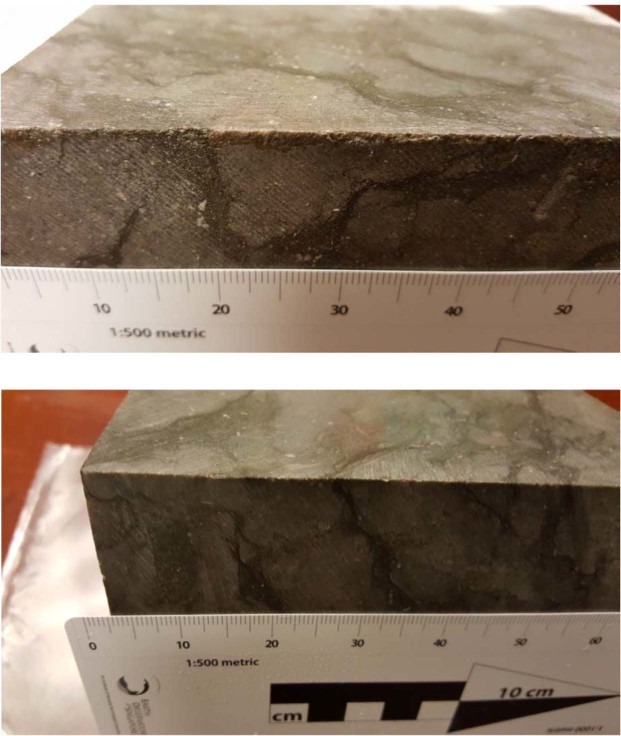
Facies continuity observed at the edge of a cuboid of Cobourg limestone.

In this paper, attention is first focused on the mapping of the spatial distribution of the lighter and darker facies of the Cobourg limestone by dissection of a cuboid measuring 80 mm × 120 mm × 300 mm into thick plate samples measuring 80 mm × 120 mm × 8 mm with a separation of 8 mm. The sizes of the lighter nodular regions tend to vary (see Figs. 1 and 2) and an average dimension is around 75 mm. The photographic images of the larger surface areas of ten thick plate sections are combined with digital imaging techniques to identify the surface features of the lighter and darker regions of each thick plate sample. The photographs were converted into binary black and white images using the *Image Processing Package* provided in MATLAB^®^. The surfaces of the samples contained a sparse distribution of fossil inclusions and striations left by the diamond saw cut that gave rise to noise in the transformed black and white images. While it was possible to use filtering algorithms to remove this noise, it was more convenient to remove the imperfections manually, using the CorelDRAW^TM^ X4 Graphics Suite. These images were used to create the plausible through-thickness fabric distributions either by a direct surface image extrusion procedure or through a solids modelling approach available in the LOFT command of the AutoCAD program, to create a three-dimensional image using replicas of surface features. (The procedures will be presented in a subsequent section.). The surface irregularities introduced during the cutting of the 80 mm × 120 mm × 300 mm cuboid could not be removed from the 8 mm thick edges of the plates without damaging of the plates. A typical view of the edges of a cube of Cobourg limestone measuring 130 mm is shown in Fig. 3. These images clearly indicate the extension of the surface fabric morphology to the interior of the domain, which needs to be accounted for in the estimation of the geomechanical properties by a composite materials approach. Following a comment by a reviewer, at the scale of the visualizations there are no issues to be addressed with regard to pore-scale modelling similar to that used in the interpretation of highly porous rocks such as sandstone (Bakke and Øren^17^), where the porosities can range from 0.1 to 0.4, whereas for the Cobourg limestone, the porosities are in the range 0.001 to 0.006. Attention is, however, drawn to the work of Klaver *et al*.^18^ dealing with pore geometries in shale, with comparable porosities.

**Figure 4:**
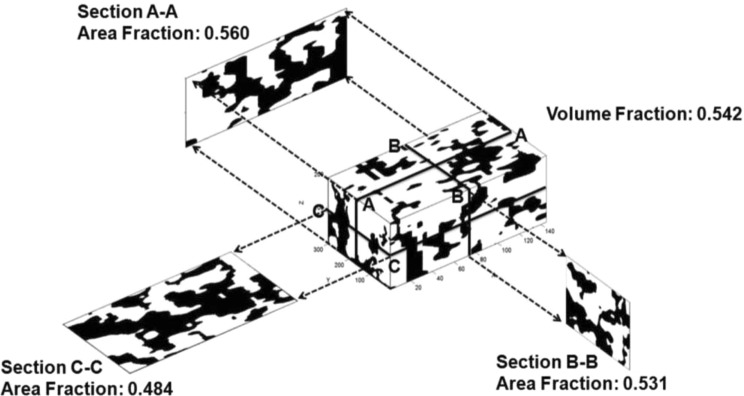
The spatial variability of the lighter and darker regions within the Cobourg limestone. [Area Fraction = *A*_DR_/*A*; Volume Fraction = *V*_DR_/*V*; *A*_DR_ and *V*_DR_ refer, respectively, to the area and volume fractions of the darker facies and *A* and *V* refer, respectively, to the total area and total volume].

The reconstructed imaging techniques of each thick plate of the Cobourg limestone are used to develop a three-dimensional replica of the plausible distribution of the lighter and darker facies of the Cobourg limestone (Fig. 4). In order to obtain a better visualization of the two facies a photographic method for recreating a 3D model of a sample block was employed. The photographs of the slabs were taken using a tripod in a fixed position. Each slab was photographed on both sides. In order to improve the contrast between the two phases of the Cobourg limestone, the photographs were taken while the surface of the sample was wet. To simulate a unique frame of reference, a mirror image was obtained from the second photographs of each sample. Each slab was then modelled to examine computationally the fluid flow through it. The facies permeabilities of the lighter and darker regions of the Cobourg limestone were used to develop the effective permeability for each thick plate in three orthogonal directions.

The effective permeability of the entire Cobourg limestone block is then estimated using both the weighted mean and the weighted harmonic mean applicable to the assemblage of ten thick plates. The weighted mean of the assemblage of the ten slabs will give the two effective in-plane permeabilities in the *x*- and *y*-directions and the weighted harmonic mean of the assemblage of the ten slabs will give the effective permeability in the *z*-direction. These estimates are necessary if an effective permeability is to be estimated through a measure such as the geometric mean. The results from the computational approach are also compared with effective permeability estimates derived from theories for the estimation of effective properties of multi-phasic composite materials^19–22^.

The estimates for the bulk permeability, as defined by the geometric mean and obtained from an effective property estimation using a composite materials approach are compared with results obtained from experimental techniques. [It should be noted that the arithmetic mean (two estimates) and harmonic mean are needed to calculate the effective permeabilities of the composite solid consisting of the ten thick slabs in the *x*, *y* and *z* directions. These are used to calculate the geometric mean. The multiphasic theories are those that (i) consider the volume fractions of the two facies and (ii) their respective isotropic permeabilities and uses the theoretical relationships proposed by Voigt^23^, Reuss^24^, Hill^19^ and Hashin and Shtrikman^20^ to arrive at an estimate for the effective permeabilities.

**Figure 5:**
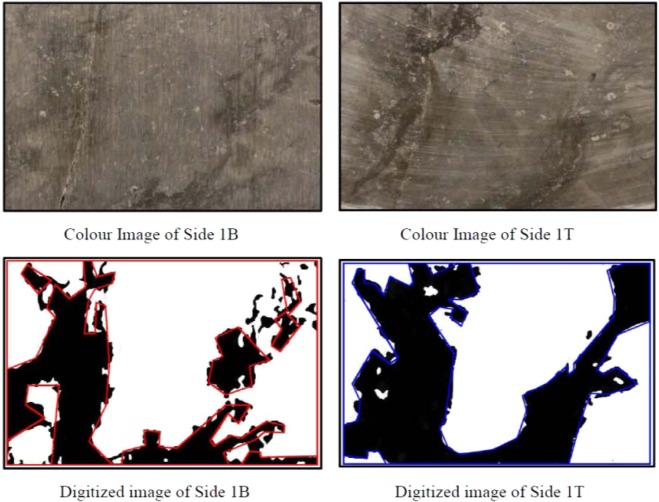
Digitized imaging of the Thick Plate Section 1 of the Cobourg limestone.

**Figure 6:**
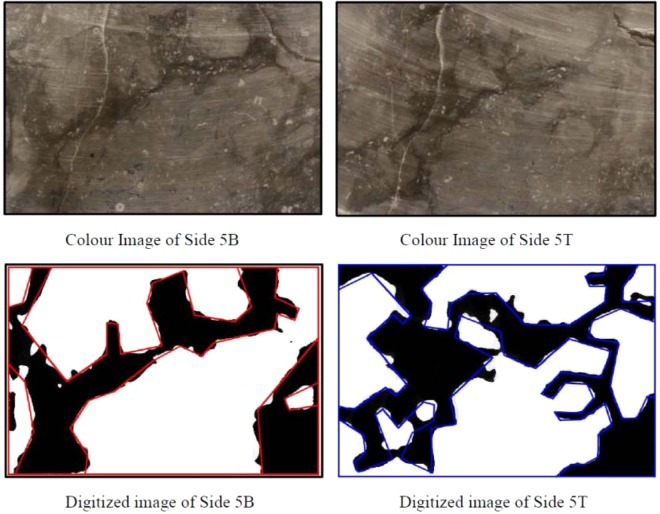
Digitized imaging of the Thick Plate Section 5 of the Cobourg limestone.

**Figure 7:**
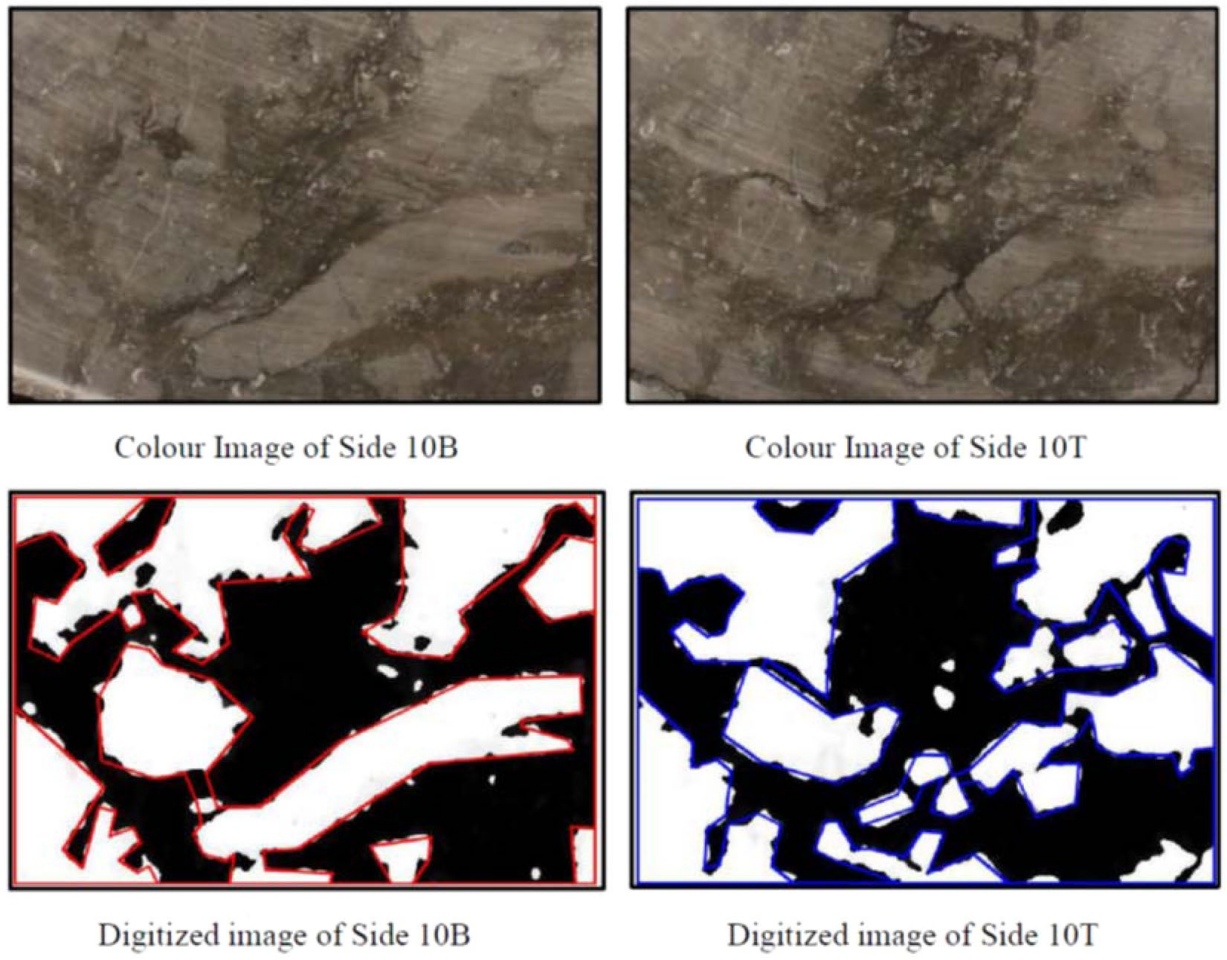
Digitized imaging of the Thick Plate Section 10 of the Cobourg limestone.

The Cobourg limestone has a visibly heterogeneous internal fabric that consists of nodular regions of a mixture of calcite and dolomite separated by argillaceous partings, which also consist of calcite and dolomite with fractions of quartz and a small clay fraction that gives the darker colour. The most effective technique for mapping the fabric is through X-ray tomographic (XRT) imaging of the rock. Such an approach was used^1^ to map the internal fabric of smaller Cobourg limestone cylinders measuring 85 mm diameter and 165 mm in length [Selvadurai^2^]. The facilities used for that particular XRT imaging had insufficient power to map the fabric of a 150 mm diameter and 300 mm long sample. The 150 mm diameter sample was dissected to discs of approximately 25 mm thickness; however, such a thickness does not allow for the accurate computer-aided reconstruction of the internal fabric of the disc. In a previous research exercise^2^, a cuboidal sample of the Cobourg limestone measuring 80 mm × 120 mm × 300 mm was saw cut into thick slabs measuring 80 mm × 120 mm × 8 mm. This sample thickness offers a better possibility for reconstructing the interior fabric of the Cobourg limestone. First, each thick plate sample was assigned a sequential position along the axis of the block. Photographic images of all the sections were prepared and catalogued according their position and orientation. Photographic images from the two plane faces of the thick sections were used to generate black and white digital images. These images identified, manually, the zones of the lighter and darker regions corresponding to the two facies of limestone encountered on the plane faces of the thick plates. Examples of the operations as applied to the thick plates of the Cobourg limestone are given in Figs. 5–7. It should also be noted that defects could be introduced during the preparation of the thick plates. These are not defects that would be present in the intact rock; the presence of such defects could result in permeabilities significantly higher than values reported in the study. The remaining task was to develop a procedure for obtaining, from the surface maps, the distribution of the lighter and darker facies within the interior of the plates.

A reviewer suggested the possibility of the numerical quantification (Heilbronner^21^) of the heterogeneity. While this is a useful line of enquiry, certainly with respect to the possible influences of *in situ* stress states and geologic heterogeneity that can be introduced by depositional effects, the value of the extension will be more meaningful in relation to a site-specific location where such a repository could be situated. The measures of quantifiable heterogeneities should ultimately be linked to hydro-mechanical properties that will be of interest to fluid transport.

## Finite Element Representations of the Fabric of the Cobourg Limestone

**Figure 8:**
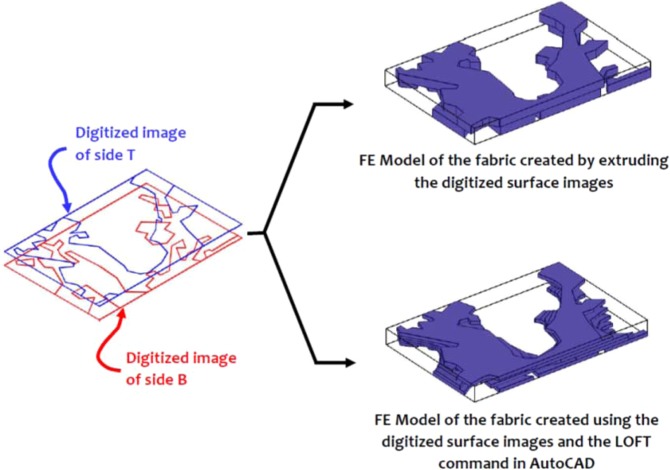
Domains for the finite element modelling of the lighter and darker facies of the Cobourg limestone in Thick Plate Section 1.

**Figure 9:**
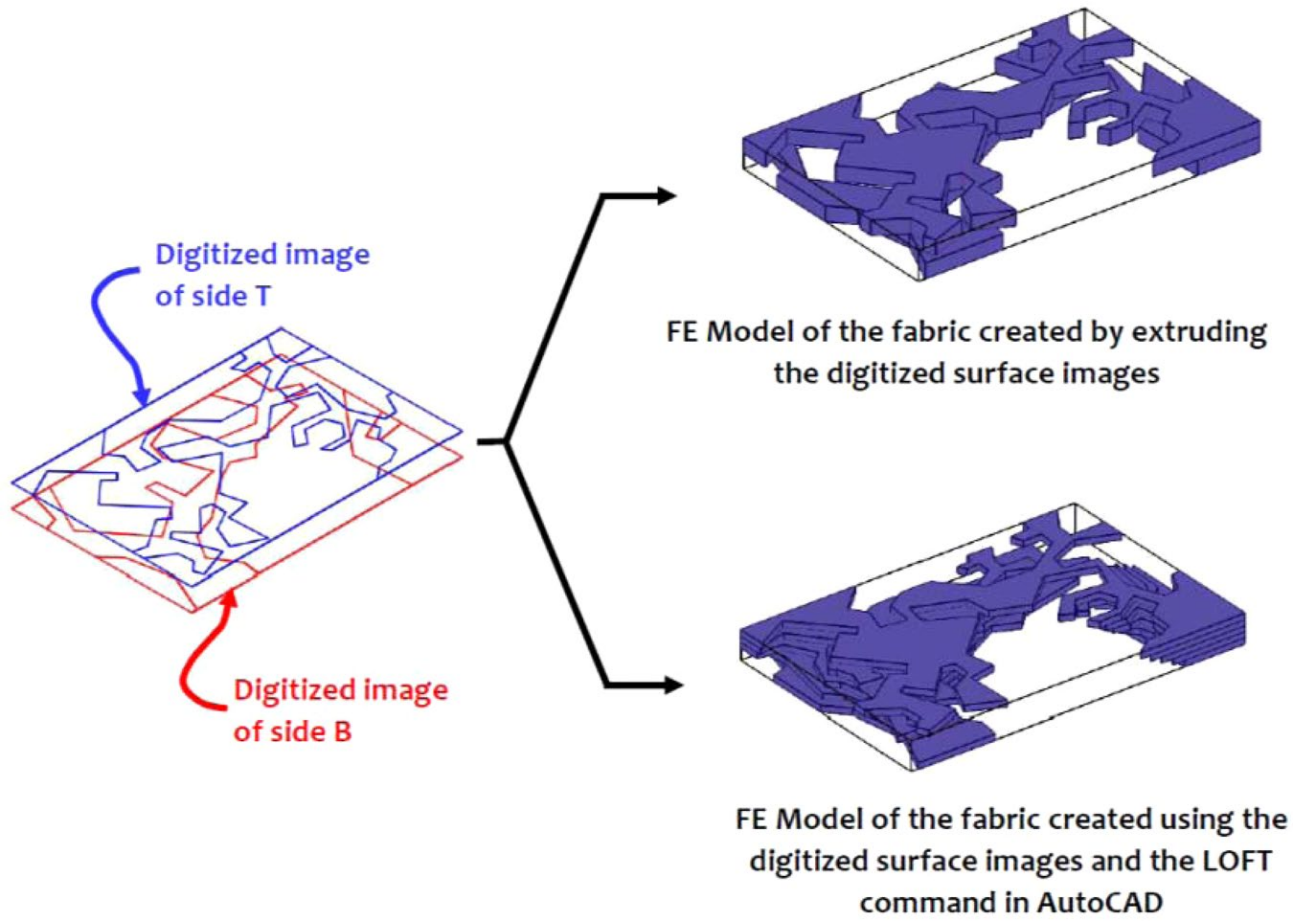
Domains for the finite element modelling of the lighter and darker facies of the Cobourg limestone in Thick Plate Section 5.

**Figure 10:**
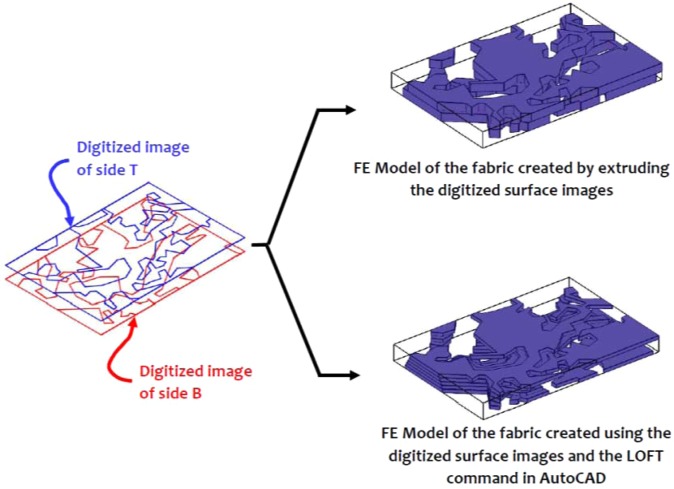
Domains for the finite element modelling of the lighter and darker facies of the Cobourg limestone in Thick Plate Section 10.

The surface imaging of the Cobourg limestone thick plates and the approximate identification of the regions of the lighter and darker facies were used to develop finite element models of each thick plate with a heterogeneous fabric. In order to perform the three-dimensional modelling of each thick plate, it was necessary to represent the fabric of the Cobourg at its interior. In this study, we adopt two approaches: in the first, the images from both surfaces of the thick plate are extruded to the mid-plane of each plate, and in the second, the LOFT command available in the AutoCAD program is used to generate the internal fabric of the lighter/darker facies using a solid modelling. In its intact condition, the Cobourg limestone is a low porosity rock with no visible presence of fractures and other defects. As briefly mentioned previously, the presence of such features in a low porosity rock would contribute to much larger values of the intact permeability compared to the values indicated in Table 1 and Table 3 of^15^. Fractures can be created during the slab cutting process and this is unavoidable. The rock contains fossil fragments and minerals that are visible but there is no evidence that these features would influence the mapping of the lighter and darker regions of the Cobourg. Due to the low porosity of the rock, attention is focused on using the LOFT command to map only the darker facies of the Cobourg, with the assumption that the remainder is the lighter region. To apply the procedure, the darker region to be used in the LOFT command should be present on both faces of the slab. In the event a darker region is not available on one face of the thick slab, a region of radius 1 mm is introduced on the face opposite to the region that will be modelled using the LOFT command. The program selects the darker region that will be modelled and creates a two-dimensional solid of specified thickness (2 mm) and fills the space between the faces with self-similar images of either increasing or decreasing dimensions. The region to be extruded need not be a closed contour. On occasions, the region to be extruded between the two faces of the slab needs to be further discretized to achieve a suitable replica of the region. It is also possible to use the kriging techniques that are available in the literature^22^ and these were successfully adopted to estimate the interior distribution of permeability for surface measurements^25,26^, but such techniques are perhaps more relevant to the re-construction of the interior distributions for general three-dimensional configurations rather than the plate-like domains that are examined in this study, and the information for the kriging process is provided from multiple surface features. In the context of modelling the interior distribution of the facies within the 8 mm thick slab of the Cobourg limestone, the LOFT command-based solids modelling approach is likely to give 3D visualizations that are expected to be similar to the approach that uses the direct extrusion of the surface features to the mid-plane of the slab. (For ease of reference, the geometry created using the LOFT command is referred to as the *Morphed* geometry.) Figs. 8–10 indicate the typical representations of the internal distribution of the lighter and darker facies in thick plate specimens 1, 5 and 10.

## Facies Permeability Estimates for the Cobourg Limestone

The successful application of any multi-phasic approach has to rely on the accurate estimation of the relevant properties of the individual phases and their respective volume fractions. The volume fractions of the lighter and darker facies of the Cobourg limestone were estimated in previous research efforts^1,2^ and the average volume fraction of the *darker* facies is variable and for the dissected cuboid it is estimated (Fig. 4) to be *V*_DR_ ≃ 0.54. [Values as low as *V*_DR_ ≃ 0.35 have also been observed^2^.]

The facies permeability is best estimated by isolating small enough samples that can be tested using one-dimensional steady state flow. This experiment requires specialized procedural techniques for ensuring interface sealing and the prevention of delaminations and defects in the samples. In the experimental technique recently proposed by Selvadurai and Głowacki^15^, miniature fluid entry ports were used to evaluate local permeability of the lighter and darker regions of a larger Cobourg limestone sample, measuring approximately 150 mm in diameter and 185 mm in length. Using miniature fluid entry ports situated in either the lighter or the darker regions, this experimental arrangement and steady state tests were conducted on both the lighter and darker regions of the Cobourg limestone to estimate the permeabilities. Table 3 in Selvadurai and Głowacki^15^ contains a range of estimates for the permeabilities of the lighter and darker facies. The permeability results from steady state tests varied from 0.27 × 10^−19^ m^2^ to 8.0 × 10^−19^ m^2^ for the darker material and 0.62 × 10^−19^ m^2^ to 6.3 × 10^−19^ m^2^ for the lighter material. The upper limit for the darker material (with higher porosity) and the lower limit for the lighter material (with lower porosity) are chosen. i.e.

1 $${K}_{{\rm{DR}}}\approx 8.00\times {10}^{-19}{{\rm{m}}}^{2};\,{K}_{{\rm{LR}}}\approx 0.62\times {10}^{-19}{{\rm{m}}}^{2}$$

The work can certainly be extended to include a range of estimated values^15^ for the respective permeabilities but the intention here is to outline a methodology that can be used as a basis for estimating the influences of heterogeneity. Also, from an applications perspective, the upper limit of permeability for the low porosity facies and the lower limit of permeability for the high porosity facies would provide a bounding of the facies permeabilities. The basic assumption is that the volume fractions can exhibit variations within the sample but the intrinsic facies permeabilities are isotropic measures and considered invariant.

## Permeability of the Cobourg Limestone

Prior to performing computations to estimate the effective permeability of the Cobourg limestone determined from the identified fabric, it is useful to estimate the effective permeability by appeal to the theory of multiphasic materials. The theories developed for estimating the effective properties such as permeability, thermal conductivity and permittivity have similar mathematical developments. Assuming that the Cobourg limestone can be modelled as a statistically homogeneous isotropic medium and provided the phasic permeabilities are non-zero, the effective permeability (K^*^) can be bounded by the Voigt^23^ and Reuss^24^ estimates in the form

2 $${[\mathop{\sum }\limits_{i=1}^{m}(\frac{{V}_{i}}{{K}_{i}})]}^{-1} < {K}^{\ast } < \mathop{\sum }\limits_{i=1}^{m}{K}_{i}{V}_{i};\,i={\rm{LR}},\,{\rm{DR}}$$

where *V*_*i*_ are the volume fractions and *K*_*i*_ are the permeabilities. Considering the permeability estimates for the lighter and darker regions given by (1) and the volume fractions *V*_*DR*_ ≃ 0.54; *V*_*LR*_ ≃ 0.46, the result (2) gives

3 $$1.235 < (\frac{{K}^{\ast }}{{10}^{-19}{{\rm{m}}}^{2}}) < 4.605$$

The bounds provide a wide but useful range for estimating the effective permeability of the Cobourg limestone. Hill^19^ provided an estimate that involves the arithmetic mean of the Voigt and Reuss bounds. This gives

4 $${(\frac{{K}^{\ast }}{{10}^{-19}{{\rm{m}}}^{2}})}_{{\rm{Hill}}}\simeq 2.920$$

The bounds can be improved by considering the theoretical developments proposed by Hashin and Shtrikman^20^ based on variational approaches, and, for a two phase system (i.e. the lighter and darker phases), takes the form

5 $${K}_{{\rm{LR}}}+\frac{{V}_{{\rm{DR}}}}{(\frac{1}{({K}_{{\rm{DR}}}-{K}_{{\rm{LR}}})}+\frac{{V}_{{\rm{LR}}}}{3{K}_{{\rm{LR}}}})}\le {K}^{\ast }\le {K}_{{\rm{DR}}}+\frac{{V}_{{\rm{LR}}}}{(\frac{1}{({K}_{{\rm{LR}}}-{K}_{{\rm{DR}}})}+\frac{{V}_{{\rm{DR}}}}{3{K}_{{\rm{DR}}}})}$$

It can be noted that when *K*_DR_ > *K*_LR_, (5) corresponds to the Wiener^25^ bounds. This result gives

6 $$2.031\le (\frac{{K}^{\ast }}{{10}^{-19}{{\rm{m}}}^{2}})\le 3.929$$

Even though the bounds do not converge to a single result, they are an improvement on the Voigt and Reuss estimates and provide a useful range for interpreting the results of the computational simulations. Improvements to these bounds can only be obtained by using mathematical approaches for estimating the effective properties of multiphasic composites where the fabric geometries are defined by appeal to specific solid features (e.g. spherical, spheroidal, ellipsoidal porous inclusion phases) embedded in a porous matrix. The internal fabric of the Cobourg limestone, however, does not lend itself to consistent mathematical modelling using geometric primitives.

The estimated internal distributions of the lighter and darker facies within each of the Cobourg limestone slabs were used to construct finite element models and these representations were used to estimate the axial and in plane permeabilities of each thick plate. The one-dimensional permeabilities of each slab in three orthogonal directions are determined in the following way: (i) by imposing constant potential Dirichlet boundary conditions, with a potential difference, on two opposite faces of the slab, and (ii) by imposing no flow or null Neumann boundary conditions on the remaining adjacent faces of the slab. This procedure is performed in all three orthogonal directions. The permeabilities of the lighter and darker phases of the Cobourg limestone can be used in the computational models of the fabric to evaluate the effective permeability values for each thick slab in (i) a direction normal to the plane of the plate and (ii) two orthogonal directions in the plane of the plate aligned with the boundaries. The computational modelling of the potential flow problem is performed using the COMSOL™ finite element code and the domains are modelled using tetrahedral elements. The number of elements for each thick plate are in excess of 150, 000 for the extruded geometry and more than 210,000 elements for the LOFT command internal fabric reconstruction. The number of degrees of freedom is generally greater than 280,000. The computational approach was used to estimate the permeabilities of ten adjacent thick plates of the Cobourg limestone obtained by dissecting the original cuboidal sample measuring 80 mm × 120 mm × 300 mm. Table 2 illustrates the estimates for the effective permeabilities obtained from the computational modelling. For ease of reference, the permeability normal to the larger surface area of the slab is denoted by *K*_*z*_ and the permeabilities for the orthogonal directions aligned with the edges of the thick slab are denoted by *K*_*x*_ and *K*_*y*_.

**Table 2:** Variation of through-plane and in-plane permeability values at the section level (*K*_LR_ = 0.62 × 10^−19^ m^2^, *K*_DR_ = 8.00 × 10^−19^ m^2^).

Plate (*i*)	Extruded Geometry			Morphed Geometry		
	${K}_{z}^{i}\,\times {10}^{-19}\,{{\rm{m}}}^{2}$	${K}_{x}^{i}\,\times {10}^{-19}\,{{\rm{m}}}^{2}$	${K}_{y}^{i}\,\times {10}^{-19}\,{{\rm{m}}}^{2}$	${K}_{z}^{i}\,\times {10}^{-19}\,{{\rm{m}}}^{2}$	${K}_{x}^{i}\,\times {10}^{-19}\,{{\rm{m}}}^{2}$	${K}_{y}^{i}\,\times {10}^{-19}\,{{\rm{m}}}^{2}$
1	1.712	2.795	2.485	1.856	2.655	2.474
2	1.753	3.106	3.063	1.619	3.106	3.090
3	1.757	2.491	2.723	2.124	2.451	2.858
4	1.833	2.847	2.199	1.876	2.623	2.130
5	1.850	2.550	2.165	1.734	1.804	1.961
6	2.232	2.629	2.112	2.107	2.312	2.022
7	2.277	2.363	2.119	2.129	1.780	1.999
8	2.221	2.872	2.059	2.263	2.227	2.153
9	2.792	3.094	2.523	2.359	1.948	2.856
10	2.671	3.850	2.435	2.954	2.797	2.545

The computational results for the 10 thick plates of the Cobourg limestone can now be combined to obtain the effective permeabilities for the assemblages. The effective permeability of the cuboid of the Cobourg limestone in the direction normal to the plane of the plates (*K*_z_^*^) can be obtained from the *Weighted Harmonic Mean*. Considering the *Extruded* geometry, we have

7 $${K}_{z}^{\ast Ext}=\frac{\mathop{\sum }\limits_{i=1}^{10}{t}_{i}}{\mathop{\sum }\limits_{i=1}^{10}(\frac{{t}_{i}}{{K}_{z}^{i}})}\simeq 2.049\times {10}^{-19}{{\rm{m}}}^{2}$$

where *t*_*i*_ are the thicknesses of the plate regions and *K*_*z*_^*i*^ are the permeabilities.

Similarly, the effective permeabilities of the cuboid in the *x* and *y* directions can be obtained from the *Weighted Mean* for the *Extruded* geometry: i.e.

8 $${K}_{x}^{\ast Ext}=\frac{\mathop{\sum }\limits_{i=1}^{10}{K}_{xi}{t}_{i}}{\mathop{\sum }\limits_{i=1}^{10}{t}_{i}};\,{K}_{y}^{\ast Ext}=\frac{\mathop{\sum }\limits_{i=1}^{10}{K}_{yi}{t}_{i}}{\mathop{\sum }\limits_{i=1}^{10}{t}_{i}};\,({\rm{no}}\,{\rm{sum}}\,{\rm{over}}\,{\rm{repeated}}\,i)$$

The estimates derived from (8) give

9 $${K}_{x}^{\ast Ext}\simeq 2.860\times {10}^{-19}{{\rm{m}}}^{2};\,{K}_{y}^{\ast Ext}\simeq 2.388\times {10}^{-19}{{\rm{m}}}^{2}$$

Similarly, considering the *Morphed* geometry, we obtain

10 $$\begin{array}{rcl}{K}_{z}^{\ast Morph} & \simeq  & 2.047\times {10}^{-19}{{\rm{m}}}^{2}\\ {K}_{x}^{\ast Morph} & \simeq  & 2.370\times {10}^{-19}{{\rm{m}}}^{2}\\ {K}_{y}^{\ast Morph} & \simeq  & 2.409\times {10}^{-19}{{\rm{m}}}^{2}\end{array}$$

It is clear that the two modes of reconstruction of the interior fabric of the Cobourg limestone yield approximately the same estimates for the effective permeabilities in the three orthogonal directions. For purposes of comparison with the Hashin-Shtrikman^20^ estimates discussed previously, we can use the Geometric Mean of the permeability estimates given by (7), (9) and (10) for the *Extruded* and *Morphed* fabric reconstructions. The geometric mean concept has been successfully applied^26,27^ to define the effective permeability of a heterogeneous cuboidal region of Indiana limestone measuring 450 mm. It is noted that the concept of the Geometric Mean in the estimation of permeability is not new. The articles^26^ and^27^, illustrate a complete treatment of the concept, which includes (i) development of a permeameter that can be used to measure the near surface permeability of the cuboidal region, (ii) a complete mathematical analysis of the permeameter performance under steady flow, (iii) the measurement of surface permeabilities at 54 locations of the cuboidal region, (iv) the development of Kriging techniques to estimate the interior permeabilities of the Indiana limestone cuboid, (v) implementation of the spatial heterogeneity of the permeability in a computational model to estimate the effective permeability of the cuboid in three orthogonal directions, (vi) estimation of the geometric mean and (vii) the use of the geometric mean concept to estimate the fluid flow in multi-dimensional configurations to validate the geometric mean concept.

For the Cobourg limestone, considering the *Extruded* fabric, we obtain

11 $${K}_{GM}^{Extr}=\sqrt[3]{({K}_{z}^{\ast Extr})({K}_{x}^{\ast Extr})({K}_{y}^{\ast Extr})}=2.410\times {10}^{-19}{{\rm{m}}}^{2}$$

Similarly, for the *Morphed* fabric, we have

12 $${K}_{GM}^{Morph}=\sqrt[3]{({K}_{z}^{\ast Morph})({K}_{x}^{\ast Morph})({K}_{y}^{\ast Morph})}=2.266\times {10}^{-19}{{\rm{m}}}^{2}$$

From these developments, it can be concluded that the effective intact permeability of the heterogeneous Cobourg limestone (K^*^) can be assigned the following bounds, assuming that the upper limit selected is the lowest computational estimate based on the geometric mean:

13 $$2.031\le (\frac{{K}^{\ast }}{{10}^{-19}{{\rm{m}}}^{2}})\le 2.266$$

## Results Based on Gas Permeability Estimates for Facies Permeabilities

The estimation of the permeability characteristics of the two phases is a challenging exercise and in the experimental techniques proposed by Selvadurai and Głowacki^15^, miniature fluid entry points were installed within either the lighter or the darker regions and both steady state flow tests and transient hydraulic pulse tests were conducted to estimate the isotropic permeabilities of the facies. The alternative was to extract samples of the lighter and darker phases and to use transient *gas permeability tests* on samples of the lighter and darker phases of the Cobourg limestone. The test procedure adopted by Cydarex^28^ involves the preparation of thin disc samples of the rock embedded in resin, with the plane ends of the disc exposed. In the tests, 2 to 5 mm thick discs were used in both steady state and transient pulse test using nitrogen at room temperature as the permeating fluid. A Klinkenberg^29^ correction was applied to estimate the permeability. The effective confining pressure applied to the sample during a permeability test was approximately 1 MPa. The *largest values* for the respective permeabilities are estimated as follows:

14 $${K}_{{\rm{DR}}}^{{\rm{G}}}\approx 0.127\times {10}^{-19}{{\rm{m}}}^{2};\,{K}_{{\rm{LR}}}^{{\rm{G}}}\approx 0.026\times {10}^{-19}{{\rm{m}}}^{2}$$

These results are *significantly lower* than the values obtained by Selvadurai and Głowacki^15^ but the results confirm the relative magnitudes of the permeabilities of the lighter and darker phases. The results obtained by Selvadurai and Glowacki^15^ are essentially for unstressed samples and a nominal reduction of the permeability with stress has been noted^30^ (an approximately 10% reduction in permeability for a 1 MPa stress change). The results shown in Table 1 do in fact suggest the possibility of obtaining experimental estimates of permeabilities in the range *K* ∈ (10^−23^, 10^−19^) m^2^. Omitting details, it can be shown that the Voigt^23^ and Reuss^24^ results for the permeability derived from gas permeability values indicated in (14) give the following bounds:

15 $$0.0456 < (\frac{{K}^{\ast }}{{10}^{-19}{{\rm{m}}}^{2}}) < 0.0805$$

The Hashin-Shtrikman^20^ bounds give the following estimates:

16 $$0.0602\le (\frac{{K}^{\ast }}{{10}^{-19}{{\rm{m}}}^{2}})\le 0.0728$$

Again, the same trends are present, in that the bounds have narrowed and they indicate improvements over the Voigt and Reuss estimates. Adopting the COMSOL^©^ finite element methodology discussed previously, the directional permeabilities for the *extruded* domains give the following estimates for the permeabilities:

17 $$\begin{array}{rcl}{K}_{z}^{\ast Ext} & \simeq  & 0.0515\times {10}^{-19}{{\rm{m}}}^{2}\\ {K}_{x}^{\ast Ext} & \simeq  & 0.0591\times {10}^{-19}{{\rm{m}}}^{2}\\ {K}_{y}^{\ast Ext} & \simeq  & 0.0547\times {10}^{-19}{{\rm{m}}}^{2}\end{array}$$

Similarly, the results for the *Morphed* fabric give the following results for the permeabilities in the three orthogonal directions:

18 $$\begin{array}{rcl}{K}_{z}^{\ast Morph} & \simeq  & 0.0511\times {10}^{-19}{{\rm{m}}}^{2}\\ {K}_{x}^{\ast Morph} & \simeq  & 0.0535\times {10}^{-19}{{\rm{m}}}^{2}\\ {K}_{y}^{\ast Morph} & \simeq  & 0.0553\times {10}^{-19}{{\rm{m}}}^{2}\end{array}$$

We can also estimate the geometric mean for the two computational estimates (17) and (18). Considering the *Extruded* fabric, we obtain

19 $${K}_{GM}^{Extr}=\sqrt[3]{({K}_{z}^{\ast Extr})({K}_{x}^{\ast Extr})({K}_{y}^{\ast Extr})}=0.0550\times {10}^{-19}{{\rm{m}}}^{2}$$

Similarly, for the *Morphed* fabric, we have

20 $${K}_{GM}^{Morph}=\sqrt[3]{({K}_{z}^{\ast Morph})({K}_{x}^{\ast Morph})({K}_{y}^{\ast Morph})}=0.0533\times {10}^{-19}{{\rm{m}}}^{2}$$

We can conclude that for the facies permeability values determined from gas permeability tests, the permeability for the intact Cobourg limestone can be assigned the Hashin-Shtrikman^20^ bounds given by (16) since the geometric mean-based results from the computational models fall outside the lower bound.

## Concluding Remarks

The estimation of the bulk permeabilities of heterogeneous porous rocks is a non-routine exercise if the rock has high spatial variability and low permeability. In the context of experimentally estimating permeability, the best approach is to conduct steady state permeability tests on samples that capture accurately the RVE of the material. For the Cobourg limestone, the sample dimensions should exceed 75 mm to account for the heterogeneity presented by the nodular nature of the rock. As the sample size increases, the steady state measurement of permeability will take an inordinate amount of time. Measurement and interpretation of permeability therefore has to use transient techniques such as hydraulic pulse tests. Even with hydraulic pulse tests, estimation of the effective compressibility of the porous skeleton, the compressibility of the solid phases constituting the porous skeleton and the porosity are non-routine experiments. The methodologies presented in the paper require the accurate estimation of the permeabilities of the lighter and darker regions of the Cobourg limestone and their respective volume fractions. In certain situations, the identification of the respective volume fractions of the lighter and darker facies can be non-routine because of the high density and low porosity of the Cobourg limestone. Conventional XRT techniques cannot be used to estimate the volume fractions of larger RVEs. The research reported uses the dissection of a cuboidal region and surface imaging techniques to map the fabric of the Cobourg limestone, which is composed of lighter and darker regions. The research draws on developments in theories of multiphasic media to obtain a set of bounds for the effective permeability. These approaches are complemented by novel techniques that re-construct, from surface imaging of the dissections, the interior distribution of the heterogeneous fabric. Computational simulations are used to determine the bulk permeability properties with the input parameters being only the phasic permeabilities. It should be noted that the modelling precludes inter-plate flow to make the computational approach more efficient. The experimental results for the phasic permeabilities can be influenced by the experimental techniques used. Any invasive technique for estimating permeability can introduce sample disturbance and the measured permeability can also be influenced by the experimental technique (e.g. interface leakage). Gas permeability measurements can also be influenced by the sample preparation procedures and the theories and corrections used to interpret the permeability (e.g. corrections for the Klinkenberg^29^ effect). An important observation of the research is that the multiphasic mathematical theories provide estimates that compare very accurately with the computational simulations. If the phasic permeabilities and their volume fractions can be accurately determined, then the effective permeability of the statistically heterogeneous rock can be determined, as a set of bounds, using a convenient analytical result. This estimation procedure also points to factors that should be addressed when conducting hydraulic pulse tests on low permeability heterogeneous porous media^30^.
